# Sample size determination for a specific region in multiregional clinical trials with multiple co-primary endpoints

**DOI:** 10.1371/journal.pone.0180405

**Published:** 2017-06-30

**Authors:** Wong-Shian Huang, Hui-Nien Hung, Toshimitsu Hamasaki, Chin-Fu Hsiao

**Affiliations:** 1 Institute of Statistics, National Chiao Tung University, Hsinchu, Taiwan; 2 Institute of Population Sciences, National Health Research Institutes, Zhunan, Miaoli County, Taiwan; 3 National Cerebral and Cardiovascular Center, Osaka, Japan; University Medical Center Gottingen, GERMANY

## Abstract

Recently, multi-regional clinical trials (MRCTs), which incorporate subjects from many countries/regions around the world under the same protocol, have been widely conducted by many global pharmaceutical companies. The objective of such trials is to accelerate the development process for a drug and shorten the drug’s approval time in key markets. Several statistical methods have been purposed for the design and evaluation of MRCTs, as well as for assessing the consistency of treatment effects across all regions with one primary endpoint. However, in some therapeutic areas (e.g., Alzheimer’s disease), the clinical efficacy of a new treatment may be characterized by a set of possibly correlated endpoints, known as multiple co-primary endpoints. In this paper, we focus on a specific region and establish three statistical criteria for evaluating consistency between the specific region and overall results in MRCTs with multiple co-primary endpoints. More specifically, two of those criteria are used to assess whether the treatment effect in the region of interest is as large as that of the other regions or of the regions overall, while the other criterion is used to assess the consistency of the treatment effect of the specific region achieving a pre-specified threshold. The sample size required for the region of interest can also be evaluated based on these three criteria.

## Introduction

Recently, global drug development has attracted much attention from pharmaceutical companies. Unlike traditional clinical trials, the design of MRCT recruiting subjects from many countries around the world under the same protocol has led to a new strategy for drug development. This kind of design has been widely adopted by global pharmaceutical companies, which seek simultaneous drug development, submission, and regulatory approval throughout key world markets to hasten the market availability of the drug, as well as improved patient access to new and innovative treatments. However, a key issue for conducting MRCTs is how to demonstrate the efficacy of a drug in all participating regions while also evaluating the possibility of applying the overall trial results to each region. To address the difficulties related to global drug development, in 1998 the International Conference on Harmonization (ICH) published “Ethnic Factors in the Acceptability of Foreign Clinical Data”, known as the E5 guideline. The idea of an MRCT was first raised in the 11th Q& A of E5 [1]. In recent years, the trend for simultaneous clinical development in the world has been rapidly rising. To establish a framework for how to demonstrate the efficacy of a drug in all participating regions while also evaluating the possibility of applying the overall trial results to each region by conducting an MRCT, the ICH released the draft E17 guideline “General principle on planning/designing Multi-Regional Clinical Trials” [2] in 2016 to describe general principles for the planning and the design of MRCTs; another aim of the work was to increase the acceptability of MRCTs in global regulatory submissions.

The Japanese Ministry of Health, Labour and Welfare issued its own guidance document on MRCTs, “Basic Principles on Global Clinical Trials” [3]. This guidance provided two methods as examples to determine the number of Japanese subjects required for establishing consistency in treatment effect between the Japanese group and the entire group. Let *D*_Japan_ and *D*_All_ represent the observed treatment effects for the Japanese group and the entire group. Method 1 in the Japanese guidance suggests that the sample size for Japan should fulfill

$$P(D_{\text{Japan}}/D_{\text{All}}>\pi )>\gamma .$$

On the other hand, suppose that an MRCT will be conducted in three regions. Let *D*_*i*_ represent the observed treatment effect for region *i*, *i* = 1,…,3. For Method 2, the sample size should be determined to satisfy

$$P(D_{1}>0,D_{2}>0,D_{3}>0)>\gamma .$$

Note that the Japanese guidance requires that π is 0.5 or greater and that γ be 0.8 or greater.

Several different statistical approaches based on Methods 1 and 2 in the Japanese guidance have been developed. Quan et al. [4] calculated the sample size required for Japan in an MRCT with normal, binary, and survival endpoints based on Method 1. Kawai et al. [5] proposed an approach, based on Method 2, to allocate the total sample size to the regions so that a high probability of observing a consistent trend under the assumed treatment effect across regions can be obtained. In addition, consistency criteria different from those of the Japan guidance have been established, such as those by Tsou et al. [6], Uesaka [7], Ko et al. [8], and Tsou et al. [9]. On the other hand, Chen et al. [10] and Huang et al. [11] considered ethnic differences and proposed methods that apply different treatment effects across regions to the design and evaluation of MRCTs.

However, most recent approaches to the design and evaluation of MRCTs are concerned with only one primary endpoint. In some therapeutic areas the clinical efficacy of a new treatment may be characterized by a set of possibly correlated endpoints, because there may be several different aspects to patients’ responses to that treatment. For example, a typical clinical trial for Alzheimer’s disease (AD) is usually conducted with cognitive, functional, and global endpoints to evaluate a symptomatic improvement in the dementia caused by the disease; the Committee for Medicinal Products for Human Use (CHMP) [12] and the Food and Drug Administration (FDA) [13] have recommended the two co-primary endpoints of these three in the development of drugs for the treatment of AD, where clinical trials with “co-primary” endpoints are designed to evaluate if the effect of a test treatment is superior (or non-inferior) to the control on all primary endpoints. Failure to demonstrate superiority on any single endpoint implies that superiority to the control treatment cannot be concluded. These endpoints are classified as follows:

1. objective cognitive tests, e.g., the AD Assessment Scale cognitive subscale(ADAS-cog) and Severe Impairment Battery (SIB);
2. self-care and activities of daily living, e.g., the AD Cooperative Study Activities of Daily Living (ADCS-ADL) and its modified version for severe AD; and
3. global assessment of change, such as the Clinician’s Interview Based Impression of Change-plus (CIBIC-plus) and the Clinical Global Impression of Improvement (CGI-I).

Having such multiple endpoints raises difficulties for statisticians in handling multiplicity in the design and analysis of clinical trials, specifically controlling Type I and Type II error rates when the endpoints are potentially correlated. When designing a trial to evaluate joint effects on all endpoints, as seen in AD clinical trials, no adjustment is needed to control the Type I error rate. However, the Type II error rate increases as the number of endpoints to be evaluated increases. This situation is referred to as “multiple co-primary endpoints” and it is related to the intersection-union problem (Hung and Wang [14]; Offen et al. [15]).In many such trials, the sample size is often unnecessarily large, which results in complications. To overcome the issue, recently many authors have discussed approaching the design and analysis of co-primary endpoints trials using fixed-sample (size) design; the extensive references in Offen et al. [15] and Sozu et al. [16] provide many examples.

In this paper, we will focus on the design and evaluation of an MRCT with multiple co-primary endpoints. As we know, the aim of an MRCT is to show the efficacy of a drug in various global regions, and concurrently to evaluate the possibility of applying the overall trial results to each region. Therefore, we will also consider the determination of the number of subjects in a specific region to establish the consistency of treatment effects between the specific region and the entire group.

This paper is organized as follows. In section 2, we demonstrate the sample size calculation for multiple endpoints with correlation. In section 3, we established three criteria to assess the consistency of treatment effects between a specific region and the entire group in MRCTs with multiple endpoints. Under each criterion, the sample size required for the region of interest is also evaluated. An example is provided in section 4. Discussions are given in section 5.

## Material and methods

### Sample size calculation

For simplicity, we focus on a most fundamental situation, where an MRCT is designed to evaluate superiority over a placebo control on *K*(≥2)continuous multiple co-primary efficacy endpoints, and the effect size for each co-primary endpoint is assumed to be uniform across *M*(≥2) regions. Consequently, we can let *X*_*ikj*_ and *Y*_*ikl*_ be efficacy responses on the *k*th co-primary endpoint for the *j*th subject and for the *l*th subject in the *i*th region receiving the test product and the placebo control, respectively, *i* = 1,…,*M*, *j* = 1,…, *N*_*i*_^T^, *l* = 1,…,*N*_*i*_^C^, and *k* = 1,…,*K*. Let **X**_*ij*_ = (*X*_*i*1*j*_, *X*_*i*2*j*_,…, *X*_*iKj*_)^*T*^ and **Y**_*il*_ = (*Y*_*i*1*l*_, *Y*_*i*2*l*_,…, *Y*_*iKl*_)^*T*^ be the outcome vectors of *K* co-primary endpoints for the *j*th subject and the *l*th subject in the *i*th region receiving the test product and the placebo control, respectively, *j* = 1,…, *N*_*i*_^T^, *l* = 1,…,*N*_*i*_^C^.

Since the effect size for each co-primary endpoint is uniform across regions, we can therefore assume that **X**_*ij*_ and **Y**_*il*_ have multivariate normal (MVN) distributions with population mean vectors $\mu^{T}=\left(\mu_{1}^{T},\ldots ,\mu_{K}^{T}\right)$ and $\mu^{C}=\left(\mu_{1}^{C},\ldots ,\mu_{K}^{C}\right)$, respectively, and a known common covariance matrix **Σ** = (*ρ*_*kk′*_*σ*_*k*_*σ*_*k′*_), where (*a*_*kk*′_) denotes the matrix whose (*k*,*k*′)^th^ element is *a*_*kk*′_, *ρ*_*kk′*_ = *corr*(*X*_*ikj*_, *X*_*ik*′*j*_) = *corr*(*Y*_*ikl*_, *Y*_*ik*′*l*_), *k* ≠ *k*′, and $\sigma_{k}^{2}=\text{Var(}X_{ikj})=\text{Var(}Y_{ikl}).$ Here, we assume that the outcome variances are known, although in actual practice, they are usually unknown and must be estimated from some data. Let $\Delta_{k}=\mu_{k}^{T}-\mu_{k}^{C}$ for *k* = 1,…*K*. Here a higher value of the population mean for each co-primary endpoint represents a better outcome. Consequently, the hypothesis testing for multiple co-primary endpoints is given as

$${\text{H}}_{0}:\Delta_{k}\leq 0\text{ for at least one k vs}{\text{. H}}_{\text{A}}:\Delta_{k}>0\text{ for all }k.$$ (1)

The null hypothesis H_0_ can be conveniently expressed as a union of a family of hypotheses. The hypothesis for each co-primary endpoint is tested at the same significance level of *α* with H_0*k*_:Δ_*k*_ ≤ 0 vs. H_A*k*_:Δ_*k*_ > 0, and the null hypothesis **H**_0_ is rejected if and only if each null hypotheses **H**_0*k*_ is rejected, so that the hypothesis testing for multiple co-primary endpoints is a test of the significance level of *α*. Although the hypothesis is one-sided, the proposed method can be straightforwardly extended to the two-sided hypothesis. Let

$${\bar{X}}_{k}=(\sum_{i=1}^{M}\sum_{j=1}^{N_{i}^{T}}X_{ikj})/\text{(}\sum_{i=1}^{M}N_{i}^{T})\text{ and }{\bar{Y}}_{k}=(\sum_{i=1}^{M}\sum_{j=1}^{N_{i}^{C}}Y_{ikj})/\text{(}\sum_{i=1}^{M}N_{i}^{C}),$$

for *k* = 1,…*K*. Also let

$$Z_{k}=\frac{{\bar{X}}_{k}-{\bar{Y}}_{k}}{\sigma_{k}\sqrt{\frac{1}{\sum_{i=1}^{M}N_{i}^{T}}+\frac{1}{\sum_{i=1}^{M}N_{i}^{C}}}},$$

for *k* = 1,…*K*. Subsequently, we will reject H_0_ at *α* level of significance if

$$Z_{k}>z_{1-\alpha }\text{ for all }k\text{,}$$

where *z*_1‒*α*_ is the 100(1-*α*) percentile of the standardized normal distribution.

Let $N^{T}=\sum_{i=1}^{M}N_{i}^{T}$ and $N^{C}=\sum_{i=1}^{M}N_{i}^{C}.$ In the design stage we assume equally sized groups, i.e., *N*^*T*^ = *N*^*C*^ = *N*. Let **Z** = (*Z*_1_,…,*Z*_*K*_)^*T*^. Then, under H_1_, **z** is distributed as an MVN with mean vector $\left(\sqrt{N/2}\right)\delta$ and covariance matrix **ρ** = (*ρ*_*kk*′_), where **δ** = (Δ_1_/*σ*_1_,…,Δ_*K*_/*σ*_*K*_)^*T*^.

Using the result in Sozu et al. [17,18], the power for rejecting the null hypothesis H_0_ can be written as

$$1-\beta =\text{Pr}\left[\overset{K}{\underset{i=1}{\cap }}\left\{Z_{k}>z_{1-\alpha }|{\text{H}}_{A}\right\}\right].$$

This power is referred to as “conjunctive power” (Senn and Bretz [19]) or “complete power” (Westfall et al. [20]). The sample size required for achieving the desired power of 1 −*β* at the significance level of *α* for the one-sided test can be found by the minimum *N* that satisfies

$$\int_{{\text{z}}_{1-\alpha }}^{\infty }\ldots \int_{{\text{z}}_{1-\alpha }}^{\infty }f\left(z_{1},\ldots ,z_{K};\left(\sqrt{N/2}\right)\delta ,\rho \right)dz_{K}\ldots \;dz_{1}\geq 1-\beta$$ (2)

where $f\left(z_{1},\ldots ,z_{K};\left(\sqrt{N/2}\right)\delta ,\rho \right)$ represents the density of MVN with mean $\left(\sqrt{N/2}\right)\delta$ and covariance matrix **ρ** corresponding to *z*_1_,…,*z*_*K*_. An iterative procedure is required to find the required sample size. The easiest way is a grid search to increase *N* gradually until the power under n exceeds the desired power of 1 − *β*, where the maximum value of the sample sizes separately calculated for each endpoint can be used as the initial values for sample size calculation. However, this often takes much computing time. To improve the speed of the sample size calculation, Sugimoto et al. [21] and Hamasaki et al.[22] provide more efficient and practical algorithms for calculating the sample sizes. Also note that since the effect size for each co-primary endpoint is assumed to be uniform across regions, there is no difference between sample size calculations for clinical trials with co-primary endpoints conducted in multiple regions and sample size calculations for clinical trials with co-primary endpoints conducted in a single region.

### Applying the results of the MRCT to a specific region

The ICH E17 says that MRCTs should investigate not only consistency in treatment effects across populations but also treatment effects in overall populations. That is, the aim of an MRCT is to show the efficacy of a drug in various global regions, and concurrently to evaluate the possibility of applying the overall trial results to each region. Suppose that we are interested in judging whether a treatment is effective in a specific region, say the *s*th region, where 1 ≤ *s* ≤ *M*. For the *k*th co-primary endpoint, let *D*_*ik*_ be the observed mean difference in the *i*th region, $D_{k}^{SC}$ be the observed mean difference from regions other than the *s*th region, and *D*_*k*_ be the observed mean difference from all regions. That is,

$$D_{ik}=(\sum_{j=1}^{N_{i}^{T}}X_{ikj})/N_{i}^{T}-(\sum_{j=1}^{N_{i}^{C}}Y_{ikj})/N_{i}^{C},$$

$$D_{k}^{SC}=(\sum_{\begin{array}{l} i=1\\ i\neq s \end{array}}^{M}\sum_{j=1}^{N_{i}^{T}}X_{ikj})/(\sum_{\begin{array}{l} i=1\\ i\neq s \end{array}}^{M}N_{i}^{T})-(\sum_{\begin{array}{l} i=1\\ i\neq s \end{array}}^{M}\sum_{j=1}^{N_{i}^{C}}Y_{ikj})/(\sum_{\begin{array}{l} i=1\\ i\neq s \end{array}}^{M}N_{i}^{C}),$$

and

$$D_{k}={\bar{X}}_{k}-{\bar{Y}}_{k}.$$

Given that the overall result is significant at the α level, we establish the following criteria to judge whether the treatment is effective in the *s*th region:

1. *D*_*s*1_ > *γ*_1_*D*_1_,…,*D*_*sK*_ > *γ*_*K*_*D*_*K*_ for 0 < *γ*_*i*_ < 1, *i* = 1,…,*K*;
2. $D_{s1}>\gamma_{1}D_{1}^{SC},\ldots ,D_{sK}>\gamma_{K}D_{K}^{SC}$ for 0 < *γ*_*i*_ < 1, *i* = 1,…,*K*;
3. *D*_*s*1_ > *h*_1_,…,*D*_*sK*_ > *h*_*K*_ for *h*_*i*_ > 0, *i* = 1,…,*K*.

Here, we can see that the first two criteria are to evaluate (i) whether the treatment effect in the region of interest is as large as that of the regions overall and (ii) of the other regions. Note that Criterion (i) assures that the estimated efficacy within a specific region is not smaller than a pre-specified portion of the global effect estimator.

When the sample size for the specific region is sufficiently large, the overall results will be dominated by the specific region. In this case, consistency is easier to be claimed with Criterion (i) than Criterion (ii). Therefore, Criterion (ii) tends to be more conservative than Criterion (i).

It should be noted that Criterion (i) is similar to Method 1 in the Japanese guidance. As indicated by Ikeda and Bretz [23], despite observing better results in both the entire population and the specific subpopulation, consistency sometimes can not be claimed with Method 1. This similar undesirable characteristics also exist for our Criterion (ii). Therefore, Ikeda and Bretz [23] suggested an alternative to Method 1. Let *p*_*s*_ denote the proportion of patients out of 2*N* in the *s*th region. If we set $h_{i}=z_{1-\phi_{i}}\sigma_{i}\sqrt{2/Np_{s}}$ for given values *ϕ*_*i*_, Criterion (iii) is similar to the alternative method established by Ikeda and Bretz [23]. Here *ϕ*_*i*_ can be thought of as the desired significant level for performing a hypothesis test for comparing the test product and the placebo control for the *i*th endpoint within patients from the specific region.

### Sample size determination for a specific region

In the design stage, once N has been determined, special consideration should be placed on the determination of the number of subjects from the specific region in the MRCT. Per ICH E17, one important issue for conducting MRCTs is that the sample size allocation of regions should be determined such that clinically meaningful differences in treatment effects among regions can be described. Since analyses of the data from a specific region in the MRCT may not have enough statistical power, the number of subjects required for the specific region should be large enough to establish the consistency of treatment effects between the specific region and the regions overall. In this regard, ICH E17 has provided five approaches that can be considered for allocating the overall sample size to regions. Briefly, the first approach is to determine the regional sample sizes such that similar trends in treatment effects across regions can be demonstrated. The second approach is to determine the sample size needed in one or more regions such that the region-specific treatment effect preserves some pre-specified proportion of the overall treatment effect. The third approach is to enrol subjects in proportion to region size. The fourth approach is to determine the regional sample sizes so that significant results within one or more regions can be achieved. The last approach is to require a fixed minimum number of subjects in one or more regions.

In this section we suggest that, similar to the second approach suggested by ICH E17, the selected sample size should satisfy that the assurance probability of the consistency criterion in (i), (ii), or (iii), given that **δ** and the overall result is significant at the α level, is maintained at a desired level, say 80%.

Let *p*_*i*_ denote the proportion of patients out of 2*N* in the *i*th region, *i* = 1,…,*M*, where $\sum_{i=1}^{M}p_{i}=1$. Also let *N*_*i*_ be the number of patients per group in the *i*th region. That is, *N*_*i*_ = *p*_*i*_*N*. The assurance probabilities of Criteria (i)–(iii), given **δ**, can be represented by

$$AP_{1}=P_{\delta }(D_{s1}>\gamma_{1}D_{1},\ldots ,D_{sK}>\gamma_{K}D_{K}|Z_{1}>z_{1-\alpha },\ldots ,Z_{K}>z_{1-\alpha }),$$

$$AP_{2}=P_{\delta }(D_{s1}>\gamma_{1}D_{1}^{SC},\ldots ,D_{sK}>\gamma_{K}D_{K}^{SC}|Z_{1}>z_{1-\alpha },\ldots ,Z_{K}>z_{1-\alpha }),$$ (3)

and

$$AP_{3}=P_{\delta }\left(D_{s1}>h_{1},\ldots ,D_{K}>h_{K}|Z_{1}>z_{1-\alpha },\ldots ,Z_{K}>z_{1-\alpha }\right),$$

Where *P*_**δ**_ is the probability measure with respect to **δ**. Here we need to determine *p*_*s*_ to ensure that the assurance probabilities of Criteria (i)–(iii) given **δ** are maintained at a desired level, say 80%. These assurance probabilities can be directly calculated by some standard normal distributions through some algebra changes; the details of the derivations of *AP*_1_–*AP*_3_ are given in S1 and S2 Files.

## Results and discussion

### Required sample sizes and assurance probabilities

Without loss of generality, we assume that we want to see whether the overall results can apply to the first region, i.e. *s* = 1. To illustrate our approach, let *K* = 2 and assume that (Δ_1_, Δ_2_) = (3,0.45) and that (*σ*_1_,*σ*_2_) = (6,1). That is, **δ** = (0.5, 0.45)^*T*^. By considering *α* = 0.025, *β* = 0.1, (*γ*_1_, *γ*_2_) = (0.5, 0.5), and *ϕ*_1_ = *ϕ*_2_ = *ϕ*, Tables 1–4 exhibit the total sample size required per group and the assurance probabilities of Criteria (i)–(iii) for *ρ*_12_ = 0.1, 0.3, 0.5, and 0.7 with various values of *p*_1_, respectively. In Table 1, the total sample size required per group for the MRCT would be 117, which is calculated from formulas (1) and (2), for *ρ*_12_ = 0.1. The first line in Table 1 indicates that if the proportion of patients out of the total number of patients in the study is 0.10, the assurance probabilities of Criteria (i) and (ii) are respectively 0.55, 0.53, while the assurance probabilities for criteria (iii) with corresponding to *ϕ* = 0.15 and *ϕ* = 0.30 are respectively 0.33 and 0.57. From Table 1, to achieve assurance probability at the 80% level, the sample size for the first region has to be around 40% of the overall sample size for criteria (i), and to be around 60% for criterion (ii). On the other hand, the assurance probabilities of Criterion (iii) will reach 80% when the values of *p*_1_ are 40% and 30% for *ϕ* = 0.15 and *ϕ* = 0.30 respectively. Note that the sample size required per group is the minimum *N* satisfying (1) and (2); therefore, the assurance probabilities for criteria (i), (ii), and (iii) must increase more if the sample size in a practical trial is larger than *N*.

**Table 1:** Sample size and assurance probabilities for observing criteria (i), (ii), and (iii) given *α* = 0.025, *β* = 0.1, (Δ_1_, Δ_2_) = (3,0.45), (*σ*_1_, *σ*_2_) = (6,1), (*γ*_1_, *γ*_2_) = (0.5, 0.5), and *ρ*_12_ = 0.1.

*p*_1_	*N*	*AP*_1_	*AP*_2_	*AP*_3_	
				*ϕ* = 0.15	*ϕ* = 0.30
0.1	117	0.5462	0.5312	0.3276	0.5683
0.2	117	0.6786	0.6368	0.5595	0.7773
0.3	117	0.7788	0.7063	0.7294	0.8901
0.4	117	0.8568	0.7539	0.8441	0.9495
0.5	117	0.9160	0.7855	0.9171	0.9793
0.6	117	0.9578	0.8033	0.9610	0.9931
0.7	117	0.9840	0.8060	0.9854	0.9985
0.8	117	0.9967	0.7855	0.9967	0.9999
0.9	117	0.9999	0.7106	0.9999	1.0000

**Table 2:** Sample size and assurance probabilities for observing criteria (i), (ii), and (iii) given *α* = 0.025, *β* = 0.1, (Δ_1_, Δ_2_) = (3,0.45), (*σ*_1_, *σ*_2_) = (6,1), (*γ*_1_, *γ*_2_) = (0.5, 0.5), and *ρ*_12_ = 0.3.

*p*_1_	*N*	*AP*_1_	*AP*_2_	*AP*_3_	
				*ϕ* = 0.15	*ϕ* = 0.30
0.1	115	0.5690	0.5549	0.3577	0.5891
0.2	115	0.6937	0.6545	0.5798	0.7861
0.3	115	0.7880	0.7198	0.7403	0.8932
0.4	115	0.8617	0.7646	0.8489	0.9502
0.5	115	0.9180	0.7944	0.9191	0.9795
0.6	115	0.9583	0.8112	0.9613	0.9931
0.7	115	0.9842	0.8135	0.9852	0.9985
0.8	115	0.9967	0.7944	0.9967	0.9999
0.9	115	0.9999	0.7238	0.9999	1.0000

**Table 3:** Sample size and assurance probabilities for observing criteria (i), (ii), and (iii) given *α* = 0.025, *β* = 0.1, (Δ_1_, Δ_2_) = (3,0.45), (*σ*_1_, *σ*_2_) = (6,1), (*γ*_1_, *γ*_2_) = (0.5, 0.5), and *ρ*_12_ = 0.5.

*p*_1_	*N*	*AP*_1_	*AP*_2_	*AP*_3_	
				*ϕ* = 0.15	*ϕ* = 0.30
0.1	114	0.5955	0.5821	0.3915	0.6142
0.2	114	0.7128	0.6761	0.6048	0.7988
0.3	114	0.8009	0.7372	0.7559	0.8991
0.4	114	0.8697	0.7790	0.8574	0.9525
0.5	114	0.9223	0.8068	0.9232	0.9804
0.6	114	0.9603	0.8224	0.9633	0.9934
0.7	114	0.9848	0.8249	0.9859	0.9985
0.8	114	0.9968	0.8069	0.9970	0.9999
0.9	114	0.9999	0.7410	0.9999	1.0000

**Table 4:** Sample size and assurance probabilities for observing criteria (i), (ii), and (iii) given *α* = 0.025, *β* = 0.1, (Δ_1_, Δ_2_) = (3,0.45), (*σ*_1_, *σ*_2_) = (6,1), (*γ*_1_, *γ*_2_) = (0.5, 0.5), and *ρ*_12_ = 0.7.

*p*_1_	*N*	*AP*_1_	*AP*_2_	*AP*_3_	
				*ϕ* = 0.15	*ϕ* = 0.30
0.1	111	0.6250	0.6125	0.4266	0.6408
0.2	111	0.7341	0.7001	0.6303	0.8123
0.3	111	0.8154	0.7566	0.7709	0.9050
0.4	111	0.8785	0.7951	0.8653	0.9551
0.5	111	0.9271	0.8206	0.9268	0.9814
0.6	111	0.9621	0.8353	0.9649	0.9936
0.7	111	0.9853	0.8370	0.9865	0.9983
0.8	111	0.9966	0.8206	0.9973	0.9999
0.9	111	0.9999	0.7603	0.9999	1.0000

In Tables 1–4, we see the following phenomena. First of all, we found that as *p*_1_ increases, the assurance probability of Criterion (i) increases. This is due to the fact that as *p*_1_ increases, the observed overall results *D*_*k*_’s will be increasingly dominated by the observed result from the first region, *D*_1*k*_’s. Secondly, we have also observed that as *p*_1_ increases, the assurance probability of Criterion (ii) increases first and then decrease later. This occurs because the observed result from regions other than the first region, $D_{k}^{SC}$‘s, is gradually dominated by *D*_1*k*_’s at first and is then completely dominated by *D*_1*k*_’s later as *p*_1_ increases. Also, the assurance probability of Criterion (iii) increases when *p*_1_ increases, since the *h*_*i*_’s decrease as *p*_1_ increases.

Another feature we observed is that *AP*_1_ > *AP*_2_ in Tables 1, 2, 3 and 4, given *p*_1_ and *γ*_*k*_. This is due to the fact that

$$\begin{array}{l} P_{\delta }\left(D_{1k}>\gamma_{k}D_{k}^{SC},\;k=1,\ldots ,K|Z_{k}>z_{1-\alpha },\;k=1,\ldots ,K\right)\\ =P_{\delta }\left(D_{1k}>\frac{\gamma_{k}}{1-p_{1}+\gamma_{k}p_{1}}D_{k},\;k=1,\ldots ,K|Z_{k}>z_{1-\alpha },\;k=1,\ldots ,K\right)\\ <P_{\delta }\left(D_{1k}>\gamma_{k}D_{k},\;k=1,\ldots ,K|Z_{k}>z_{1-\alpha },\;k=1,\ldots ,K\right) \end{array}$$

since

$$\begin{array}{l} 0<1-p_{1}+\gamma_{k}p_{1}\\ =1-(1-\gamma_{k})p_{1}\\ <1. \end{array}$$

Like Ikeda and Bretz [23] suggested, for Criterion (iii), it may be able to link the choice of *ϕ* to (*γ*_1_, *γ*_2_) = (0.5, 0.5) in order to ensure the same level of strictness of Criterion (i). For example, in Table 1, setting *ϕ* = 0.17 in Criterion (iii) would closely ensure a similar level of strictness as Criterion (i) with *p*_1_ = 0.4. Another point we wish to make is that the assurance probabilities of all criteria increase as *ρ*_12_ increases. This makes intuitive sense because these two co-primary endpoints look more alike.

### Numerical example

In this section, we provide an example to illustrate a practical application of our method. A randomized, double-blind, active-controlled MRCT will be conducted in patients with mild to moderate AD for comparing a new treatment and a placebo control. In this trial, patients age 50 or older with a diagnosis of uncomplicated AD are planned to be recruited from three regions: Taiwan, the European Union, and the United States. The primary endpoints are the change from baseline of ADAS-cog at week 24 and the CIBIC plus value at week 24. Based on the results observed in a previous exploratory study, the differences of change in ADAS-cog score from the baseline and the CIBIC-plus at week 24 between the test drug and placebo are expected to be 2.88 and 0.44, respectively. Also the standard deviations for both groups for change in ADAS-cog score from the baseline and the CIBIC-plus at week 24 are respectively equal and are assumed to be 6.15 and 0.92. With *ρ*_12_ = 0, 0.3, 0.5, 0.8, *α* = 0.025, and *β* = 0.1, the sample sizes required per group determined by (1) and (2) are as follows:

$$n=\left\{ \begin{array}{l} \text{116 if }\rho_{12}\text{= 0,}\\ \text{114 if }\rho_{12}\text{= 0.3,}\\ \text{112 if }\rho_{12}\text{= 0.5,}\\ 107\text{ if }\rho_{12}\text{= 0.8.} \end{array}\right.$$

In addition, in order to demonstrate an overall treatment effect from all regions the sponsor is also interested in assessing whether the overall results from the multi-regional trial can be bridged to Taiwan if the overall treatment effect shows statistical significance. In this regard, the proportion of the patients recruited in Taiwan needs to be determined during the design phase of the trial to preserve the probability of establishing consistency between Taiwan and all other regions. Suppose that similarity criterion (i) is used, and that *γ*_1_ = 0.5 and *γ*_2_ = 0.5 are chosen. To insure the assurance probability of *AP*_1_ at the 80% level, the sample sizes required per group, *n*_S_, from Taiwan patients with respect to *ρ*_12_ = 0, 0.3, 0.5, and 0.8 are shown below:

$$n_{S}=\left\{ \begin{array}{l} 116\times 33\%\approx 39\text{ or }40\text{ if }\rho_{12}=0,\\ 114\times 32\%\approx 37\text{ or }3\text{8 if }\rho_{12}=0.3,\\ 112\times 30\%\approx 34\text{ if }\rho_{12}=0.5,\\ 1\text{07}\times \text{27}\%\approx \text{2}9\text{ or 3}0\text{ if }\rho_{12}=0.\text{8.} \end{array}\right.\;$$

## Conclusions

The aim of an MRCT is to show the efficacy of a drug in various global regions, and simultaneously to evaluate the possibility of applying the overall trial results to each region. However, in MRCTs sponsors are challenged by how to demonstrate consistency between a specific region and the overall results. In this paper, three criteria have been established to assess the similarity between a specific region and the overall regions in an MRCT with multiple co-primary endpoints. Regulators and sponsors can easily adopt these criteria to conduct statistical assessments of the consistency of treatment effects between the specific region and the entire trial, and consequently to help registration of the new drug in the specific region.

On the other hand, the 11th Q&A for ICH E5 states, “It may be desirable in certain situations to achieve the goal of bridging by conducting a multi-regional trial under a common protocol that includes sufficient numbers of patients from each of multiple regions to reach a conclusion about the effect of the drug in all regions.” Therefore, the sample size determination for each region is another challenge for regulators and sponsors. With the three criteria we established, the sample size required for a specific region can easily be determined so that there is a high probability of observing a consistent trend in treatment effect between the specific region and the entire MRCT. In this paper, we do not particularly recommend any criterion for evaluating the consistency of treatment effects between the entire region and the specific region.

Although our approach is easy to use, the selection of the magnitude *γ*_*i*_’s consistency trend raises an important issue. In this regard, the Japanese guidance suggests that the magnitude be 0.5 or greater for the first criterion when the number of primary endpoints for the MRCT is only one. Our suggestion is that the determination of *γ*_*i*_ should be discussed between the regulatory agency in the specific region and the trial sponsor. Most importantly, all differences in race, diet, environment, culture, and medical practice among regions should be considered.

It should be noted that, in our approach, the sample size calculation for the specific region did not have a closed-form expression. For conducting an MRCT with only one primary endpoint, Ikeda and Bretz [23] discussed the methods proposed in the Japanese regulatory guidance document and derived closed-form expressions for the resulting probabilities, which required the evaluation of multivariate normal or *t* probabilities between the overall effect and the effect in Japan. In addition, they proposed a different method of calculating the probability of observing a consistent trend based on Method 1 in the Japanese regulatory guidance. Ikeda and Bretz’s work is worthy of being extended to the MRCT with multiple co-primary endpoints.

When more than one primary endpoint is viewed as important in a clinical trial, a decision must be made as to whether it is desirable to evaluate the joint effects on at least one or even all of the endpoints. This decision defines the alternative hypothesis to be tested and provides a framework for trial design. This article discusses only the former situation, where a trial is designed to evaluate the joint effects of a new treatment compared to any control treatment on all of the primary endpoints as seen in AD clinical trials. On the other hand, the latter situation—i.e., designing the trial to evaluate an effect on at least one of the primary endpoints is referred to as “multiple primary endpoints” (Offen et al. [15])—and many methods for dealing with such multiple primary endpoints have been proposed (e.g., see the extensive references in Dmitrienko et al [24]). Similarly, as in multiple co-primary endpoints, the power for detecting an effect on at least one endpoint—which is called “disjunctive power” (Senn and Bretz [19]) or “minimal power” (Westfall et al. [20]))—can be defined and extended.

Another issue we want to point out is that in this paper, it is assumed that the outcome variances are known for the sample size calculation. In actual practice, the outcome variances are not known and should be estimated from some data. In fact, extensive literature of results of similar trials may exist, and thus the variability associated with the primary endpoints can also be found in literature. For methods for unknown variance, the major change is that the power function will be evaluated based on a non-central multivariate t-distribution. For clinical trials with multiple co-primary endpoints, Sozu et al. [18] discussed a method for the unknown variance case and showed that the calculated sample size is nearly equivalent to that for the known variance in the setting of 80% or 90% power at 2.5% significance level for one-sided test. They showed that the sample size per group calculated using the method based on the unknown variance needs generally one more subject than that using the method based on the known variance. This is a very similar result observed as in a single primary endpoint case. Therefore, sample size calculation based on a known variance provides a reasonable approximation for the unknown variances case.

Similarly, the correlation is usually unknown and thus must be estimated by (1) using data from pilot studies or proceeding clinical trials (e.g., Phase II trials), or by (2) borrowing information from external existing data when incorporating correlation into sample size calculation. In some disease areas, the correlations among the endpoints have been known. For example, Offen et al. [15] provides a list of known disease are as that the regulatory agency requires for co-primary endpoints when evaluating the effects of a new treatment; the list includes possible correlations among endpoints for each disease area.

The proposed criteria can be extended from one to multiple regions. For example, after the MRCT has demonstrated a statistically significant overall treatment effect, we can bridge the results of the MRCT to all regions if

$$D_{i1}>\gamma_{k}^{i}D_{1},\ldots ,D_{iK}>\gamma_{K}^{i}D_{K}\text{for }0<\gamma_{k}^{i}<1,\;i=1,\ldots ,M,\;k=1,\ldots ,\;K.$$

Here $\gamma_{k}^{i}$ represents the threshold of consistency trend for the *k*th endpoint in the *i*thregion. Our research work here assumes that the effect size for each co-primary endpoint and the correlations among endpoints are both uniform across regions. Since MRCTs recruit subjects from many countries around the world, it might be expected that there is a difference in treatment effect or in correlations among endpoints due to regional difference (e.g., ethnic difference).Thus, the sample size calculation for MRCTs based on the assumption that the effect size for each co-primary endpoint and the correlations among endpoints are uniform across regions might be impractical. Future work is being pursued to address this issue.

## Supporting information

S1 File Derivations of the three assurance probabilities *AP*_1_, *AP*_2_, and *AP*_3_. (PDF)

S2 File Codes of R software for *AP*_1_, *AP*_2_, and *AP*_3_. (PDF)
