# Supplementary material for: Sample size determination for a specific region in multiregional clinical trials with multiple co-primary endpoints
Source: PLoS One. 2017 Jun 30;12(6):e0180405. doi: 10.1371/journal.pone.0180405 (PMC5493407; doi:10.1371/journal.pone.0180405)
Supplement: S1 File — (PDF) [file pone.0180405.s001.pdf]

S1 File. Derivation of the four assurance probabilities AP_1, AP_2, and AP_3.

The derivation of the assurance probabilities for the sth specific region is given in detail below.

To derive AP_1, we have

$$P_{\boldsymbol{\delta}}\left(D_{s1} > \gamma_1 D_1, ..., D_{sK} > \gamma_K D_K\right)$$

$$= P_{\boldsymbol{\delta}}\left(D_{sk} > \gamma_k D_k,\ k = 1, ..., K\right)$$

$$= P_{\boldsymbol{\delta}}\left(D_{sk} > \gamma_k \sum_{i=1}^{M} p_i D_{ik},\ k = 1, ..., K\right)$$

$$= P_{\boldsymbol{\delta}}\left(\left(1 - \gamma_k p_s\right) D_{sk} - \sum_{\substack{i=1 \\ i \neq S}}^{M} \gamma_k p_i D_{ik} > 0,\ k = 1, ..., K\right)$$

$$= P_{\boldsymbol{\delta}}\left(\left(1 - \gamma_k p_s\right) \sigma_k \sqrt{\frac{2}{N_s}} \frac{D_{sk}}{\sigma_k \sqrt{\frac{2}{N_s}}} - \sum_{\substack{i=1 \\ i \neq S}}^{M} \gamma_k p_i \sigma_k \sqrt{\frac{2}{N_i}} \frac{D_{ik}}{\sigma_k \sqrt{\frac{2}{N_i}}} > 0,\ k = 1, ..., K\right)$$

$$= P_{\boldsymbol{\delta}}\left(\left(1 - \gamma_k p_s\right) \sigma_k \sqrt{\frac{2}{N_s}} \left(\frac{D_{sk} - \Delta_k}{\sigma_k \sqrt{\frac{2}{N_s}}} + \frac{\Delta_k}{\sigma_k \sqrt{\frac{2}{N_s}}}\right)\right.$$

$$\left. - \sum_{\substack{i=1 \\ i \neq S}}^{M} \gamma_k p_i \sigma_k \sqrt{\frac{2}{N_i}} \left(\frac{D_{ik} - \Delta_k}{\sigma_k \sqrt{\frac{2}{N_i}}} + \frac{\Delta_k}{\sigma_k \sqrt{\frac{2}{N_i}}}\right) > 0,\ \ k = 1, ..., K\right)$$

$$=P_0\left((1-\gamma_k p_s)\sigma_k\sqrt{\frac{2}{N_s}}\left(Z_{Sk}+\frac{\Delta_k}{\sigma_k\sqrt{\frac{2}{N_s}}}\right)\right.$$

$$\left.-\sum_{\substack{i=1\\i\neq S}}^{M}\gamma_k p_i\sigma_k\sqrt{\frac{2}{N_i}}\left(Z_{ik}+\frac{\Delta_k}{\sigma_k\sqrt{\frac{2}{N_i}}}\right)>0,\ k=1,\ldots,K\right)$$

$$=P_0\left(c_{sk}Z_{sk}-\sum_{\substack{i=1\\i\neq s}}^{M}c_{ik}Z_{ik}>\left(-(1-\gamma_k p_s)\Delta_k\right)+\sum_{\substack{i=1\\i\neq s}}^{M}\gamma_k p_i\Delta_k,\forall\ k=1,\ldots,K\right)$$

$$=P_0\left(c_{sk}Z_{sk}-\sum_{\substack{i=1\\i\neq s}}^{M}c_{ik}Z_{ik}>c_{sk}^{*},\ k=1,\ldots,K\right)$$

where

$$c_{sk}=(1-\gamma_k p_s)\sigma_k\sqrt{\frac{2}{N_s}},\ c_{ik}=\gamma_k p_i\sigma_k\sqrt{\frac{2}{N_i}}\ ,$$

and

$$c_{sk}^{*}=\left(-(1-\gamma_k p_s)\Delta_k\right)+\sum_{\substack{i=1\\i\neq s}}^{M}\gamma_k p_i\Delta_k,\ \ k=1,\ldots,K\ .$$

Furthermore, we can obtain that

$$P_{\boldsymbol{\delta}}(Z_k>z_{1-\alpha},\ k=1,\ldots,K)$$

$$=P_{\boldsymbol{\delta}}\left(\frac{1}{\sigma_k\sqrt{\frac{2}{N}}}\sum_{i=1}^{M}p_iD_{ik}>z_{1-\alpha},\ k=1,\ldots,K\right)$$

$$=P_{\boldsymbol{\delta}}\left(\sum_{i=1}^{M}p_iD_{ik}>\sigma_k\sqrt{\frac{2}{N}}z_{1-\alpha},\ k=1,\ldots,K\right)$$

$$= P_{\boldsymbol{\delta}}\left(\sum_{i=1}^{M}\left(p_i\sigma_k\sqrt{\frac{2}{N_i}}\left(\frac{D_{ik}-\Delta_k}{\sigma_k\sqrt{\frac{2}{N_i}}}+\frac{\Delta_k}{\sigma_k\sqrt{\frac{2}{N_i}}}\right)\right)>\sigma_k\sqrt{\frac{2}{N}}z_{1-\alpha},\ k=1,\ldots,K\right)$$

$$= P_0\left(\sum_{i=1}^{M}\left(p_i\sigma_k\sqrt{\frac{2}{N_i}}\left(Z_{ik}+\frac{\Delta_k}{\sigma_k\sqrt{\frac{2}{N_i}}}\right)\right)>\sigma_k\sqrt{\frac{2}{N}}z_{1-\alpha},\ k=1,\ldots,K\right)$$

$$= P_0\left(\sum_{i=1}^{M}\left(p_i\sigma_k\sqrt{\frac{2}{N_i}}Z_{ik}\right)>\sigma_k\sqrt{\frac{2}{N}}z_{1-\alpha}-\sum_{i=1}^{M}p_i\Delta_k,\ k=1,\ldots,K\right)$$

$$= P_0\left(\sum_{i=1}^{M}\left(p_i\sqrt{\frac{2}{N_i}}Z_{ik}\right)>\sqrt{\frac{2}{N}}z_{1-\alpha}-\sum_{i=1}^{M}\frac{p_i\Delta_k}{\sigma_k},\ k=1,\ldots,K\right)$$

$$= P_0\left(\sum_{i=1}^{M}p_i^{*}Z_{ik}>c_k^{**},\ k=1,\ldots,K\right)$$

where

$$p_i^{*}=p_i\sqrt{\frac{2}{N_i}}\ \text{and}\ c_k^{**}=\sqrt{\frac{2}{N}}z_{1-\alpha}-\sum_{i=1}^{M}\frac{p_i\Delta_k}{\sigma_k},\quad k=1,\ldots,K.$$

Consequently, it follows that

$$AP_1 = P_{\boldsymbol{\delta}}\left(D_{s1}>\gamma_1 D_1,\ldots,D_{sK}>\gamma_K D_K \mid Z_1>z_{1-\alpha},\ldots,Z_K>z_{1-\alpha}\right)$$

$$=\frac{P_{\boldsymbol{\delta}}\left(D_{s1}>\gamma_1 D_1,\ldots,D_{sK}>\gamma_K D_K, Z_1>z_{1-\alpha},\ldots,Z_K>z_{1-\alpha}\right)}{P_{\boldsymbol{\delta}}\left(Z_1>z_{1-\alpha},\ldots,Z_K>z_{1-\alpha}\right)}$$

$$=\frac{P_0\left(c_{sk}Z_{sk}-\sum_{\substack{i=1\\i\neq s}}^{M}c_{ik}Z_{ik}>c_{sk}^{*},\ \sum_{i=1}^{M}p_i^{*}Z_{ik}>c_k^{**},\quad k=1,\ldots,K\right)}{P_0\left(\sum_{i=1}^{M}p_i^{*}Z_{ik}>c_k^{**},\quad k=1,\ldots,K\right)}$$

$$=\frac{P_0\left(W_k>c_{sk}^{*},\ V_k>c_k^{**},\quad k=1,\ldots,K\right)}{P_0\left(V_k>c_k^{**},\quad k=1,\ldots,K\right)},$$

where

$$W_k = c_{sk} Z_{sk} - \sum_{\substack{i=1 \\ i \neq s}}^{M} c_{ik} Z_{ik}$$

and

$$V_k = \sum_{i=1}^{M} p_i^* Z_{ik},$$

for $k = 1, ..., K$. It can be seen that both W_k and V_k are linear combinations of random variables with normal distribution. Denote $\mathbf{W} = (W_1, ..., W_K)^T$ and $\mathbf{V} = (V_1, ..., V_K)^T$. Therefore, the vector including $\mathbf{W}$ and $\mathbf{V}$ will follow an MVN distribution with mean $\boldsymbol{\delta}_{\mathbf{W}}$ and $\boldsymbol{\delta}_{\mathbf{V}}$, and covariance $\boldsymbol{\Sigma}_{\mathbf{W}}$, $\boldsymbol{\Sigma}_{\mathbf{V}}$, and $\boldsymbol{\Sigma}_{\mathbf{W},\mathbf{V}}$. That is,

$$\begin{bmatrix} \mathbf{W} \\ \mathbf{V} \end{bmatrix} \sim N_{2K}\left(\begin{bmatrix} \boldsymbol{\delta}_{\mathbf{W}} \\ \boldsymbol{\delta}_{\mathbf{V}} \end{bmatrix}, \begin{bmatrix} \boldsymbol{\Sigma}_{\mathbf{W}} & \boldsymbol{\Sigma}_{\mathbf{W},\mathbf{V}} \\ \boldsymbol{\Sigma}_{\mathbf{V},\mathbf{W}} & \boldsymbol{\Sigma}_{\mathbf{V}} \end{bmatrix} \right),$$

where

$$\boldsymbol{\delta}_{\mathbf{W}} = E(\mathbf{W}) = \mathbf{0}, \ \boldsymbol{\delta}_{\mathbf{V}} = E(\mathbf{V}) = \mathbf{0},$$

and the covariance for $\mathbf{W}$ is $\boldsymbol{\Sigma}_{\mathbf{W}}$, in which the (k,k)th element is

$$\begin{aligned} \operatorname{cov}(W_k, W_{k'}) &= \operatorname{cov}\left(c_{sk} Z_{sk} - \sum_{\substack{i=1 \\ i \neq s}}^{M} c_{ik} Z_{ik},\ c_{sk'} Z_{sk'} - \sum_{\substack{i=1 \\ i \neq s}}^{M} c_{ik'} Z_{ik'} \right) \\ &= \operatorname{cov}(c_{sk} Z_{sk}, c_{sk'} Z_{sk'}) + \sum_{\substack{i=1 \\ i \neq s}}^{M} c_{ik} c_{ik'} \operatorname{cov}(Z_{ik}, Z_{ik'}) \\ &= \left(c_{sk} c_{sk'} + \sum_{\substack{i=1 \\ i \neq s}}^{M} c_{ik} c_{ik'} \right) \rho_{kk'}, \end{aligned}$$

and the covariance for $\mathbf{V}$ is $\boldsymbol{\Sigma}_{\mathbf{V}}$, in which the (k,k)th element is

$$\operatorname{cov}(V_k, V_{k'}) = \operatorname{cov}\left(\sum_{i=1}^{M} p_i^* Z_{ik}, \sum_{i=1}^{M} p_i^* Z_{ik'} \right)$$

$$= \sum_{i=1}^{M} \left(p_i^* \right)^2 \operatorname{cov}\left(Z_{ik}, Z_{ik'} \right)$$

$$= \rho_{kk'} \left(\sum_{i=1}^{M} \left(p_i^* \right)^2 \right),$$

and the covariance for $\mathbf{W}$ and $\mathbf{V}$ is $\boldsymbol{\Sigma}_{\mathbf{W},\mathbf{V}}$, whose the $(k,k)^{\text{th}}$ element is

$$\operatorname{cov}(W_k, V_{k'}) = \operatorname{cov}\left(c_{sk} Z_{sk} - \sum_{\substack{i=1 \\ i \neq s}}^{M} c_{ik} Z_{ik},\ \sum_{i=1}^{M} p_i^* Z_{ik'} \right)$$

$$= \operatorname{cov}\left(c_{sk} Z_{sk}, p_S^* Z_{sk'} \right) - \sum_{\substack{i=1 \\ i \neq s}}^{M} c_{ik} p_S^* \operatorname{cov}\left(Z_{ik}, Z_{ik'} \right)$$

$$= \left(c_{sk} p_s^* - \sum_{\substack{i=1 \\ i \neq s}}^{M} c_{ik} p_i^* \right) \rho_{kk'},$$

for $k, k' = 1, \ldots, K$. We can therefore calculate the probability AP_1 by using the distribution of $(\mathbf{W}, \mathbf{V})^T$.

With respect to AP_2, we have

$$AP_2 = P_{\boldsymbol{\delta}} \left(D_{s1} > \gamma_1 D_1^{SC}, \ldots, D_{sK} > \gamma_K D_K^{SC} \mid Z_1 > z_{1-\alpha}, \ldots, Z_K > z_{1-\alpha} \right)$$

$$= P_{\boldsymbol{\delta}} \left(D_{sk} > \gamma_k D_k^{SC},\ k = 1, \ldots, K \mid Z_k > z_{1-\alpha},\ k = 1, \ldots, K \right).$$

Note that D_k^{SC} can be re-expressed as follows:

$$D_k^{SC} = \sum_{\substack{i=1 \\ i \neq s}}^{M} \sum_{j=1}^{n_i} \frac{X_{kij}}{N - N_s} - \frac{Y_{kij}}{N - N_s}$$

$$= \sum_{\substack{i=1 \\ i \neq s}}^{M} \frac{N_i}{N - N_s} \sum_{j=1}^{n_i} \frac{X_{kij}}{N_i} - \frac{Y_{kij}}{N_i}$$

$$= \frac{N}{N-N_s}\sum_{\substack{i=1\\i\neq s}}^{M}\frac{N_i}{N}\sum_{j=1}^{n_i}\frac{X_{kij}}{N_i}-\frac{Y_{kij}}{N_i}$$

$$= \frac{N}{N-N_s}\sum_{\substack{i=1\\i\neq s}}^{M} p_i D_{ik}$$

$$= \frac{N}{N-N_s}\left(\sum_{i=1}^{M} p_i D_{ik} - p_s D_{sk}\right)$$

$$= \frac{N}{N-N_s}\sum_{i=1}^{M} p_i D_{ik} - \frac{N}{N-N_s} p_s D_{sk}$$

$$= \frac{N}{N-N_s} D_k - \frac{N}{N-N_s} p_s D_{sk}$$

$$= \frac{N}{N-N_s} D_k - \frac{N_s}{N-N_s} D_{sk}$$

This leads to

$$P_{\boldsymbol{\delta}}\left(D_{sk} > \gamma_k D_k^{SC},\ k=1,\dots,K \mid Z_k > z_{1-\alpha},\ k=1,\dots,K\right)$$

$$= P_{\boldsymbol{\delta}}\left(D_{sk} > \gamma_k\left(\frac{N}{N-N_s}D_k - \frac{N_s}{N-N_s}D_{sk}\right),\ k=1,\dots,K \mid Z_k > z_{1-\alpha},\ k=1,\dots,K\right)$$

$$= P_{\boldsymbol{\delta}}\left(D_{sk} + \gamma_k\frac{N_s}{N-N_s}D_{sk} > \gamma_k\frac{N}{N-N_s}D_k,\ k=1,\dots,K \mid Z_k > z_{1-\alpha},\ k=1,\dots,K\right)$$

$$= P_{\boldsymbol{\delta}}\left(\left(\frac{N-N_s+\gamma_k N_s}{N-N_s}\right)D_{sk} > \gamma_k\frac{N}{N-N_s}D_k,\ k=1,\dots,K \mid Z_k > z_{1-\alpha},\ k=1,\dots,K\right)$$

$$= P_{\boldsymbol{\delta}}\left(D_{sk} > \gamma_k\frac{N}{N-N_s+\gamma_k N_s}D_k,\ k=1,\dots,K \mid Z_k > z_{1-\alpha},\ k=1,\dots,K\right)$$

$$= P_{\boldsymbol{\delta}}\left(D_{sk} > \gamma_k\frac{1}{1-p_s+\gamma_k p_s}D_k,\ k=1,\dots,K \mid Z_k > z_{1-\alpha},\ k=1,\dots,K\right)$$

$$= P_{\boldsymbol{\delta}}\left(D_{sk} > \gamma'_k D_k,\ k=1,\dots,K \mid Z_k > z_{1-\alpha},\ k=1,\dots,K\right)$$

where

$$\gamma'_k = \gamma_k \frac{1}{1 - p_s + \gamma_k p_s}.$$

The rest of the derivation of AP_2 is then similar to the derivation of AP_1 above with γ_k replaced by γ'_k, for $k = 1, ..., K$.

To derive AP_3, we have

$$P_{\boldsymbol{\delta}}\left(D_{sk} > h_k, k = 1, ..., K\right) = P_{\boldsymbol{\delta}}\left(\frac{D_{sk}}{\sigma_k\sqrt{\frac{2}{N_s}}} > \frac{h_k}{\sigma_k\sqrt{\frac{2}{N_s}}}, k = 1, ..., K\right)$$

$$= P_{\boldsymbol{\delta}}\left(\frac{D_{sk} - \Delta_k}{\sigma_k\sqrt{\frac{2}{N_s}}} > \frac{h_k - \Delta_k}{\sigma_k\sqrt{\frac{2}{N_s}}}, k = 1, ..., K\right)$$

$$= P_{\mathbf{0}}\left(\mathrm{Z}_{sk} > \frac{h_k - \Delta_k}{\sigma_k\sqrt{\frac{2}{N_s}}}, k = 1, ..., K\right)$$

$$= P_{\mathbf{0}}\left(\mathrm{Z}_{sk} > h_k^*, k = 1, ..., K\right),$$

where

$$h_k^* = \frac{h_k - \Delta_k}{\sigma_k\sqrt{\frac{2}{N_s}}}$$

Thus, it follows that

$$AP_3 = P_{\boldsymbol{\delta}}\left(D_{s1} > h_1, ..., D_{sK} > h_K \mid Z_1 > z_{1-\alpha}, ..., Z_K > z_{1-\alpha}\right)$$

$$= \frac{P_{\boldsymbol{\delta}}\left(D_{s1} > h_1, ..., D_{sK} > h_K, Z_1 > z_{1-\alpha}, ..., Z_K > z_{1-\alpha}\right)}{P_{\boldsymbol{\delta}}\left(Z_1 > z_{1-\alpha}, ..., Z_K > z_{1-\alpha}\right)}$$

$$= \frac{P_{\mathbf{0}}\left(Z_{sk} > h_k^*, \quad \sum_{i=1}^{M} p_i^* Z_{ik} > c_k^{**}, \quad k = 1, \ldots, K \right)}{P_{\mathbf{0}}\left(\sum_{i=1}^{M} p_i^* Z_{ik} > c_k^{**}, \quad k = 1, \ldots, K \right)}.$$

We therefore obtain the AP_3 in a similar way to AP_1 by replacing the h_k^* with c_{sk}^* and setting $c_{sk} = 1$ and $c_{ik} = 0$, $i \neq s$.
