# Supplementary material for: Sample size determination for a specific region in multiregional clinical trials with multiple co-primary endpoints
Source: PLoS One. 2017 Jun 30;12(6):e0180405. doi: 10.1371/journal.pone.0180405 (PMC5493407; doi:10.1371/journal.pone.0180405)
Supplement: S2 File — (PDF) [file pone.0180405.s002.pdf]

S2 File. Codes of R software for AP_1, AP_2, and AP_3.

```
#S2 Program Code. Codes of R software for AP1, AP2, AP3, and AP4.
#We assume the specific region is the first region.
#Note that the accuracy control such as maxpts, abseps, and releps in the "mvtnorm" package
would influence the accuracy when the number of endpoints increases.

#-----Parameter settings-----#
rm(list=ls(all=TRUE))
alpha <- 0.025      # input the alpha
beta <- 0.1         # input the beta
M <- 3              # input the number of regions
pre_rho <- 0.7      # input the correlation rho between endpoints
K <- 2              # number of endpoints.

# input gamma_K
ga_K <- c(0.5,0.5)


# input the delta values each co-primary endpoint
delta_K <- c(3, 0.45)


# input the sigma values each co-primary endpoint
sigma_K <- c(6, 1)

# input the phi for Iketa and Bretz's method
phi=0.15

#install.packages("mvtnorm")
library("mvtnorm")


##
AP_above <- function(K){
      p <- 2*K
      lower <- c(c_star_K,c_star2_K)
      upper <- rep(Inf,p)
```

```
	t  <-  pmvnorm(lower=lower, upper=upper, mean=rep(0,p), sigma=B_sigma)
	return(t)
}

####### Calculate the denominator of AP
AP_denominator <- function(K){
	p <- K
	lower <- c(c_star2_K)
	upper <- rep(Inf,p)
	t  <-  pmvnorm(lower=lower, upper=upper, mean=rep(0,p), sigma=B_sigma_V)
	return(t)
}

##### construct the correlation matrix
rho <- matrix(NA,K,K)
for(i in 1:K){
	for(j in 1:K){
		if(i==j){
		   rho[i,j] <- 1}else{
		   rho[i,j] <- pre_rho
		}
	}
}

res <- c(NA)


time <- Sys.time()
p_v <- seq(0.1,0.9,0.1)
L_pp <- length(p_v)
res1 <- matrix(NA,L_pp,2)
for(pp in 1:L_pp){
	p_specific <- p_v[pp]
	delta_M_K <- matrix(rep(delta_K,M),M,K,byrow=TRUE)
	ap_decom <- rep(NA,1)
	q <- qnorm(1-alpha)
	p_M <- c(p_specific,rep((1-p_specific)/(M-1),M-1))
```

```
### Calculate the required sample size
## initial value for searching the required sample sizes
N <- ceiling((((qnorm(1-beta)+qnorm(1-alpha))/min(delta_K/sigma_K))^2) # initial value by traditional method
go <- 0

while(N<10000 && go==0){
    N_M <- p_M*N
    p_star <- p_M*sqrt(2/N_M)
    c_star2_K <- rep(NA,K)
    for(m in 1:K){
        c_star2_K[m] <- sqrt(2/N)*q-sum(p_M*delta_M_K[,m]/sigma_K[m] )
    }

    B_sigma_V <- matrix(NA,K,K)
    for(i in 1:K){
        for(j in 1:K){
            if(i==j){
                B_sigma_V[i,j] <- sum(p_star^2)
            }else{
                B_sigma_V[i,j] <- sum(p_star^2)*rho[i,j]
            }
        }
    }
    power <- AP_denominator(K)
    if(power>(1-beta)) {go <- 1}
    #print.table(c(N,power))
    N <- N+1
}
N <- N-1
N_M <- p_M*N
p_star <- p_M*sqrt(2/N_M)

for(AP_v in 1:2){
    APchoose <- AP_v    # select the number of APs
    r_K_AD <- ga_K
    if(APchoose==1) {AD <- 1} # AD for adaption. AD=1 for AP1 and AP2; AD=4 for AP3 and
```

AP4

```
    if(APchoose==2) {AD <- 1;r_K_AD <- r_K_AD*N/(N-N_M[1]+r_K_AD*N_M[1])}

# h=1 for AP1 and AP2 with adjusted gamma
    for(h in 1:AD){
        if(h==1) {r_K <- r_K_AD}

        B_sigma_W <- matrix(NA,K,K)
        B_sigma_W_V <- matrix(NA,K,K)



        c_v_K <- matrix(NA,M,K)
        c_K <- rep(NA,K)
        for(r in 1:M){
            if(r==1)
            {
                for(m in 1:K)
                {c_v_K[r,m] <- (1-r_K[m]*p_M[r])*sigma_K[m]*sqrt(2/N_M[r])}
            }else{
                for(m in 1:K)
                {c_v_K[r,m] <- r_K[m]*p_M[r]*sigma_K[m]*sqrt(2/N_M[r])}
            }

        }

        c_star_v_K <- matrix(NA,M,K)
        c_star_K <- rep(NA,K)
        for(r in 1:M){
            if(r==1)
            {
                for(m in 1:K)
                {c_star_v_K[r,m] <- (-(1-r_K[m]*p_M[r]))*delta_M_K[r,m]}
            }else{
                for(m in 1:K)
                {c_star_v_K[r,m] <- r_K[m]*p_M[r]*delta_M_K[r,m]}
            }
        }
```

```
	for(m in 1:K)
	{c_star_K[m] <- sum(c_star_v_K[,m])}

	for(i in 1:K){
		for(j in 1:K){
			if(i==j)
			{
				c_ex <- 0
				for(r in 1:M){
					if(r==1)
					{
						c_s <- c_v_K[r,i]*c_v_K[r,j]
					}else{
						c_ex <- c_ex+c_v_K[r,i]*c_v_K[r,j]
					}
				}
				B_sigma_W[i,j] <- c_s+c_ex
			}else{
				c_ex <- 0
				for(r in 1:M){
					if(r==1)
					{
						c_s <- c_v_K[r,i]*c_v_K[r,j]
					}else{
						c_ex <- c_ex+c_v_K[r,i]*c_v_K[r,j]
					}
				}
			B_sigma_W[i,j] <- (c_s+c_ex)*rho[i,j]
		}
	}
}


for(i in 1:K){
	for(j in 1:K){
		if(i==j){
			c_p_ex <- 0
			for(r in 1:M){
```

```
                    if(r==1)
                    {
                        c_p_s <- c_v_K[r,i]*p_star[r]
                    }else{
                        c_p_ex <- c_p_ex+c_v_K[r,i]*p_star[r]
                    }

                }
                B_sigma_W_V[i,j] <- c_p_s-c_p_ex
            }else{
                c_p_ex <- 0
                for(r in 1:M){
                    if(r==1){
                        c_p_s <- c_v_K[r,i]*p_star[r]
                    }else{
                        c_p_ex <- c_p_ex+c_v_K[r,i]*p_star[r]
                    }
                }
                B_sigma_W_V[i,j] <- (c_p_s-c_p_ex)*rho[i,j]
            }
        }

    }
        B_sigma <-
rbind(cbind(B_sigma_W,B_sigma_W_V),cbind(t(B_sigma_W_V),B_sigma_V))
        AP_a <- AP_above(K)
        ap_decom[h] <- AP_a
    }   #end of h

        AP_b <- power
        AP <- AP_a/AP_b
        res1[pp,AP_v] <- AP
    } # end of AP_v
    res <- rbind(res,c(alpha,beta,p_specific,N,res1[pp,]))
} # end of pp
res <- res[-1,]
res
```

```
##### AP_IB after obtaining the threhold h
res.IB <- c(NA)
p_v <- seq(0.1,0.9,0.1)
L_pp <- length(p_v)
res1.IB <- matrix(NA,L_pp,1)
for(pp in 1:L_pp){
	p_specific <- p_v[pp]
	delta_M_K <- matrix(rep(delta_K,M),M,K,byrow=TRUE)
	ap_decom <- rep(NA,1)
	q <- qnorm(1-alpha)
	p_M <- c(p_specific,rep((1-p_specific)/(M-1),M-1))


	### Calculate the required sample size
	## initial value for searching the required sample sizes
	N <- ceiling(((qnorm(1-beta)+qnorm(1-alpha))/min(delta_K/sigma_K))^2) # initial value by 
traditional method
	go <- 0

	while(N<10000 && go==0){
		N_M <- p_M*N
		p_star <- p_M*sqrt(2/N_M)
		c_star2_K <- rep(NA,K)
		for(m in 1:K){
			c_star2_K[m] <- sqrt(2/N)*q-sum(p_M*delta_M_K[,m]/sigma_K[m] )
		}

		B_sigma_V <- matrix(NA,K,K)
		for(i in 1:K){
			for(j in 1:K){
				if(i==j){
					B_sigma_V[i,j] <- sum(p_star^2)
				}else{
					B_sigma_V[i,j] <- sum(p_star^2)*rho[i,j]
				}
			}
```

```
        }
        power <- AP_denominator(K)
        if(power>(1-beta)) {go <- 1}
        #print.table(c(N,power))
        N <- N+1
}
N <- N-1
N_M <- p_M*N
p_star <- p_M*sqrt(2/N_M)


N_specific=(p_specific*N)
z.phi=qnorm(1-phi)
thre.h=z.phi*sigma_K*sqrt(2/N_specific)

h.star.K=(thre.h-delta_K)/(sigma_K*sqrt(2/N_specific))
h_star_K=h.star.K
delta_M_K <- matrix(rep(delta_K,M),M,K,byrow=TRUE)
q <- qnorm(1-alpha)
p_M <- c(p_specific,rep((1-p_specific)/(M-1),M-1))

        N_M <- p_M*N
        p_star <- p_M*sqrt(2/N_M)
        c_star2_K <- rep(NA,K)
        for(m in 1:K){
                c_star2_K[m] <- sqrt(2/N)*q-sum(p_M*delta_M_K[,m]/sigma_K[m] )
        }

        B_sigma_V <- matrix(NA,K,K)
        for(i in 1:K){
                for(j in 1:K){
                        if(i==j){
                                B_sigma_V[i,j] <- sum(p_star^2)
                        }else{
                                B_sigma_V[i,j] <- sum(p_star^2)*rho[i,j]
                        }
                }
        }
```

```
        power <- AP_denominator(K)

N_M <- p_M*N
p_star <- p_M*sqrt(2/N_M)

APchoose <- 1    # select the number of APs
r_K_AD <- ga_K
r_K <- r_K_AD

        B_sigma_W <- matrix(NA,K,K)
        B_sigma_W_V <- matrix(NA,K,K)

        c_v_K <- matrix(NA,M,K)
        c_K <- rep(NA,K)
        for(r in 1:M){
                if(r==1)
                {
                        for(m in 1:K)
                        {c_v_K[r,m] <- 1}

                }else{
                        for(m in 1:K)
                        {c_v_K[r,m] <- 0}

                }

        }

        c_star_v_K <- matrix(NA,M,K)
        c_star_K <- rep(NA,K)
        for(m in 1:K)
        {c_star_K[m] <- h_star_K[m]}

        for(i in 1:K){
                for(j in 1:K){
                        if(i==j)
                        {
                                c_ex <- 0
```

```
                for(r in 1:M){
                    if(r==1)
                    {
                        c_s <- c_v_K[r,i]*c_v_K[r,j]
                    }else{
                        c_ex <- c_ex+c_v_K[r,i]*c_v_K[r,j]
                    }
                }
                B_sigma_W[i,j] <- c_s+c_ex
            }else{
                c_ex <- 0
                for(r in 1:M){
                    if(r==1)
                    {
                        c_s <- c_v_K[r,i]*c_v_K[r,j]
                    }else{
                        c_ex <- c_ex+c_v_K[r,i]*c_v_K[r,j]
                    }
                }
            B_sigma_W[i,j] <- (c_s+c_ex)*rho[i,j]
        }
    }
}


for(i in 1:K){
    for(j in 1:K){
        if(i==j){
            c_p_ex <- 0
            for(r in 1:M){
                if(r==1)
                {
                    c_p_s <- c_v_K[r,i]*p_star[r]
                }else{
                    c_p_ex <- c_p_ex+c_v_K[r,i]*p_star[r]
                }

            }
```

```
                    B_sigma_W_V[i,j] <- c_p_s-c_p_ex
                }else{
                    c_p_ex <- 0
                    for(r in 1:M){
                        if(r==1){
                            c_p_s <- c_v_K[r,i]*p_star[r]
                        }else{
                            c_p_ex <- c_p_ex+c_v_K[r,i]*p_star[r]
                        }
                    }
                    B_sigma_W_V[i,j] <- (c_p_s-c_p_ex)*rho[i,j]
                }
            }

        }
            B_sigma <-
rbind(cbind(B_sigma_W,B_sigma_W_V),cbind(t(B_sigma_W_V),B_sigma_V))
            AP_a <- AP_above(K)
            AP_b <- power
            AP.IB <- AP_a/AP_b
            res1.IB[pp,] <- AP.IB
        res.IB <- rbind(res.IB,c(alpha,beta,p_specific,N,res1.IB[pp,]))
} # end of pp

res.IB=res.IB[-1,]
output=cbind(res,phi, res.IB[,5])
dimnames(output) <- list(NULL,c('alpha','beta','p_s','N','AP1','AP2','Phi','AP.IB'))
output
pre_rho
#write.table((output),"D:/ress.txt",sep="\t",col.name=FALSE,row.name=FALSE,append=TRUE)
```
